# Plasma oxytocin levels in response to glucagon in patients with arginine vasopressin deficiency (central diabetes insipidus) and healthy controls

**DOI:** 10.1007/s12020-024-03920-2

**Published:** 2024-06-27

**Authors:** Cihan Atila, Shalini Mekkattu, Rakithan Murugesu, Odile Gaisl, Nimmy Varghese, Anne Eckert, Mirjam Christ-Crain

**Affiliations:** 1 Department of Endocrinology, Diabetology and Metabolism, University Hospital Basel, Basel, Switzerland; 2 Department of Clinical Research, University Hospital Basel, University of Basel, Basel, Switzerland; 3 Psychiatric University Hospital, University of Basel, Basel, Switzerland; 4 Research Cluster, Molecular & Cognitive Neuroscience, Division of Neurobiology, University of Basel, 4002 Basel, Switzerland

**Keywords:** Diabetes insipidus, Oxytocin, Glucagon test, Hypopituitarism, Pituitary, Stimulation test

## Abstract

**Purpose:**

We recently demonstrated an additional oxytocin (OT) deficiency in patients with arginine vasopressin (AVP) deficiency (central diabetes insipidus) by using 3,4-methylenedioxy-methamphetamine (MDMA) as a novel provocation test. However, the implication of the MDMA provocation test in clinical practice might be challenging. Glucagon effectively stimulates vasopressinergic neurons with a strong increase in plasma copeptin. We therefore hypothesized that this provocation test might also stimulate OT.

**Methods:**

This is a predefined secondary analysis of a prospective double-blind, randomised, placebo-controlled cross-over trial involving ten patients with AVP deficiency and ten sex- and body-mass index-matched healthy participants at the University Hospital Basel, Switzerland. Each participant underwent the glucagon test (s.c. injection of 1 mg glucagon) and placebo test (s.c. injection of 0.9% normal saline). Plasma OT levels were measured at baseline, 60, 120 and 180 min after injection. The primary objective was to determine whether glucagon stimulates OT and whether OT levels differ between patients with AVP deficiency and healthy participants. The primary outcome (maximum change in OT within 180 min) was compared between groups and conditions using a linear mixed effects model.

**Results:**

In healthy participants, the median OT at baseline was 82.7 pg/ml [62.3–94.3] and slightly increased to a maximum of 93.3 pg/ml [87.2–121.1] after injection of glucagon, resulting in a change increase of 24.9 pg/ml [5.1–27.8]. Similarly, in patients with AVP deficiency, the median OT at baseline was 73.9 pg/ml [65.3–81.6] and slightly increased after glucagon injection to 114.9 pg/ml [70.9–140.9], resulting in a change increase of 36.8 pg/ml [–2.2 to 51.2]. The results from the mixed model showed no effect between glucagon compared to placebo on OT (difference: –0.5 pg/ml; 95%-CI [–25, 24]; *p* = 0.97) and no significant treatment-by-group interaction effect between patients compared to healthy participants (interaction: 28 pg/ml; 95%-CI [–7, 62]; *p* = 0.13).

**Conclusion:**

We found no effect of glucagon on plasma OT levels and no difference between patients with AVP deficiency and healthy participants.

## Introduction

Oxytocin (OT) and arginine vasopressin (AVP) are neuropeptides that are synthesised in the same hypothalamic paraventricular and supraoptic nuclei and released into circulation from the posterior pituitary [1]. OT is, among others, involved in regulating labour and stimulating lactation as well as in psychological and socio-behavioural processes [2–4].

Central diabetes insipidus, also known as AVP deficiency, is a neuroendocrine disorder resulting from disturbances of the hypothalamic-pituitary axis leading to impaired osmo-regulated secretion of AVP [5]. Patients with AVP deficiency often experience psychological problems such as heightened anxiety, depressed mood, sleep difficulties and reduced sexual drives as well as lower quality of life, despite adequate desmopressin (AVP receptor 2 analogue) treatment [6]. These psychosocial symptoms, along with the shared anatomical pathways of synthesis and secretion of AVP and OT, suggest an increased likelihood of additional OT deficiency in patients suffering from AVP deficiency [7]. Indeed, in our recent study, we demonstrated an OT deficiency in these patients by using 3,4-methylenedioxy-methamphetamine (MDMA) as a provocation test [8]. Although this strong provocation test provides the advantages of both psychoactive and biochemical stimulation, its implication in clinical practice might be challenging. Therefore, a more simplified, safe provocation test would be of great clinical importance. Recently, we provided evidence supporting glucagon as an effective stimulating agent of AVP-producing neurons with a strong increase in plasma copeptin as a surrogate marker of AVP. The suggested mechanism of action is the rapid decrease of elevated plasma glucose in response to glucagon mimicking a hypoglycaemia-like stimulus without absolute hypoglycemia [9]. Previously, some studies have indicated that hypoglycaemia induced by the insulin hypoglycaemia test also leads to OT release [10, 11]. Therefore, considering that OT is produced by the same neuron population sensitive to glucose changes, we hypothesised that glucagon might also stimulate OT secretion. Thus, this current analysis aims to determine whether glucagon stimulates OT secretion and whether OT levels differ between patients with AVP deficiency and healthy participants.

## Material and methods

### Trial design

This is a predefined secondary analysis of a prospective single-centre, double-blind, randomised, placebo-controlled, cross-over trial conducted at the University Hospital Basel, Switzerland, from September 2020 to May 2021. The study protocol was approved by the Ethics Committee Northwest Switzerland, and all study participants provided written informed consent. The study was registered on ClinicalTrials.gov with the identifier NCT 04550520.

### Participants

This analysis involved a subset of the original study and used data from ten patients with AVP deficiency and ten sex- and body mass index (BMI)-matched healthy participants. The study details are available elsewhere [12]. Briefly, healthy participants were eligible for inclusion if they were 18 years or older, had a BMI between 18.5 kg/m^2^ and 25 kg/m^2^, normal drinking habits (<3 l fluid intake in 24 h) and had no history of polyuria. Participants were excluded if they showed evidence of altered drinking habits (i.e., polydipsia: >3 l/24 h) and diuresis (i.e., polyuria: >40 ml/kg body weight/ 24 h and/or >3 l/24 h), had a haemoglobin level below 120 g/l, were acutely ill, or had engaged in vigorous physical exercise within 24 h of the study. Patients with AVP deficiency were diagnosed based hypertonic saline infusion test, some patients were additionally tested with the water-deprivation or arginine stimulation test, respectively. Patients were eligible for inclusion if they were 18 years or older, had a BMI between 18.5 kg/m^2^ and 25 kg/m^2^, and were taking regular daily desmopressin medication. Exclusion criteria were haemoglobin level below 120 g/l, acute illness, and vigorous physical exercise 24 h before the study participation.

### Study procedure

Each participant completed two test days, one receiving a subcutaneous injection of 1 mg glucagon and the other receiving a 1 ml injection of placebo (0.9% sodium chloride), in random order with a minimum of 48 h between the two days. The injection contents were unknown to the study team and participants. Two unblinded study nurses administered the injection but were not involved in further study procedures. The test day began between 8:00 and 10:00 a.m. after an overnight meal fast of 8 h, during which fluid intake was restricted for the last 2 h. Participants were not allowed to drink during the test procedure. Patients taking desmopressin treatment discontinued their medication at least 24 h before the test. Participants were placed in a semi-recumbent position 30 min before the test began, and a venous catheter was inserted. All participants received oral ondansetron 8 mg 10 min before the test to prevent nausea. At baseline, the first blood sample was collected, and then glucagon 1 mg (Glucagen® NovoNordisk – Hypokit) or placebo (0.9% sodium chloride) was injected. Blood samples were collected at baseline, 60, 120 and 180 min for plasma copeptin and OT. Furthermore, growth hormone, prolactin, and cortisol were measured at time points 0 and 150 min.

### Laboratory measurements

Blood samples were collected in EDTA tubes for plasma OT analysis, immediately centrifuged at 4 °C at 3000 rpm for 10 min, and stored at –80 °C until central batch analysis. Duplicate measurements of extracted plasma OT were taken in one batch for the glucagon stimulation test using an enzyme-linked immunosorbent assay (ELISA) kit (ENZO Life Sciences, Ann Arbor, MI) with a sensitivity of 15 pg/ml (range 15.6–1000 pg/ml). In brief, plasma was diluted in TFA-H2O, and the collected supernatant was loaded onto an Oasis PRiME HLB 96-well plate, 30 mg sorbent (Waters Corporation, Milford, MA). The sample was eluted with 95% acetonitrile + 5% of a 0.1% TFA-H2O solution. The reconstituted samples, standards, and controls were plated on the goat anti-Rabbit IgG microtiter plate and incubated with OT conjugate overnight. The plate was read at 405 nm, and the OT concentration was calculated using a standard curve calculated from the 4-parameter logistics curve fit. The intra-assay coefficient of variation for our OT is 1.59%, and the inter-assay coefficient of variation is 4.97%. The antiserum cross-reacts with mesotocin of 7%, arginine vasotocin of 7.5%, and <0.02% for other related molecules. Plasma copeptin concentration was measured in one batch with a commercial automated immunofluorescence assay (B.R.A.H.M.S Copeptin-proAVP KRYPTOR, Thermo Scientific Biomarkers, Hennigsdorf, Germany), prolactin, growth hormone and cortisol levels were measured using an electrochemiluminescence immunoassay (ECLIA).

### Study outcomes and statistical analysis

Demographic information and laboratory parameters were described using median [IQR] or absolute (relative) frequency as appropriate. The primary endpoint of this analysis was to evaluate the maximum increase in plasma OT levels within three hours following a single subcutaneous injection of either 1 mg glucagon or 1 ml 0.9% sodium chloride. To determine the primary outcome (i.e., OT^∆^glucagon and OT^∆^placebo) for each participant and trial arm, the maximal OT value between 60 and 180 min after injection (OT_max_) was calculated, and the corresponding baseline measurement was subtracted (OT_baseline_). The continuous primary endpoint was compared between the two treatments and participant groups using linear mixed-effects regression models with a random intercept for each participant. The main predictor of interest was the interaction between treatment (placebo over glucagon) and group (patient over healthy participants), and the baseline value of OT was included as a covariate. Secondary endpoints included the maximal changes of growth hormone, prolactin, and cortisol. Correlations between glucose vs. OT or copeptin levels were assessed by calculating Spearman’s rank correlation coefficient. A correlation coefficient (Spearman’s rho) of 0.00–0.40 was considered a weak correlation, 0.41-0.60 as moderate, and 0.61-1.00 a strong correlation [13]. All statistical analyses were executed using R version 4.0.3 and were exploratory without hypothesis testing.

### Role of funding source

The study was not influenced by its funders in design, data collection, analysis, interpretation, or report writing.

## Results

### Baseline characteristics

In healthy participants, the median [IQR] age was 25 years [23–28] [14, 15] with 60% female participants, and in patients with AVP deficiency, the median age was 31 years [27–44] [16], with 60% female participants. In patients, eight had complete and two had partial AVP deficiency. The underlying aetiologies were idiopathic (*n* = 3), genetic/hereditary (*n* = 3), Rathke’s cleft cyst/craniopharyngioma (*n* = 2), and inflammatory/autoimmune (*n* = 2). Baseline characteristics are presented in Table 1.

**Table 1:** Baseline characteristics

	Healthy controls	Patients with AVP-deficiency
Number	10	10
Sex, female	6 (60)	6 (60)
Ethnicity, Caucasian	9 (90)	9 (90)
Ethnicity, Non-Caucasian	1 (10)	1 (10)
Age, years	25 [23, 28]	31 [27, 44]
Weight, kg	62 [59, 67]	66 [63, 72]
Height, cm	171 [163, 175]	170 [166, 176]
BMI, kg/m^2^	22.6 [20.6, 24.5]	22.1 [21.2, 22.9]
Degree in AVP deficiency, complete	NA	8 (80)
Aetiology of AVP deficiency		
Idiopathic	NA	3 (30)
Genetic/hereditary	NA	3 (30)
Rathke’s cleft cyst/craniopharyngioma	NA	2 (20)
Inflammatory/autoimmune	NA	2 (20)
Anterior pituitary insufficiency*	0 (0)	3 (30)
Adrenocorticotropic hormone	0 (0)	2 (20)
Thyrotropic hormone	0 (0)	2 (20)
Growth hormone	0 (0)	3 (30)
Gonadotropins	0 (0)	3 (30)

Data are presented in absolute (relative) frequency and median [IQR]

*List includes only tested and diagnosed ones

*AVP* arginine vasopressin, *NA* not applicable

### Oxytocin and copeptin levels after glucagon and placebo stimulation

**Fig. 1:**
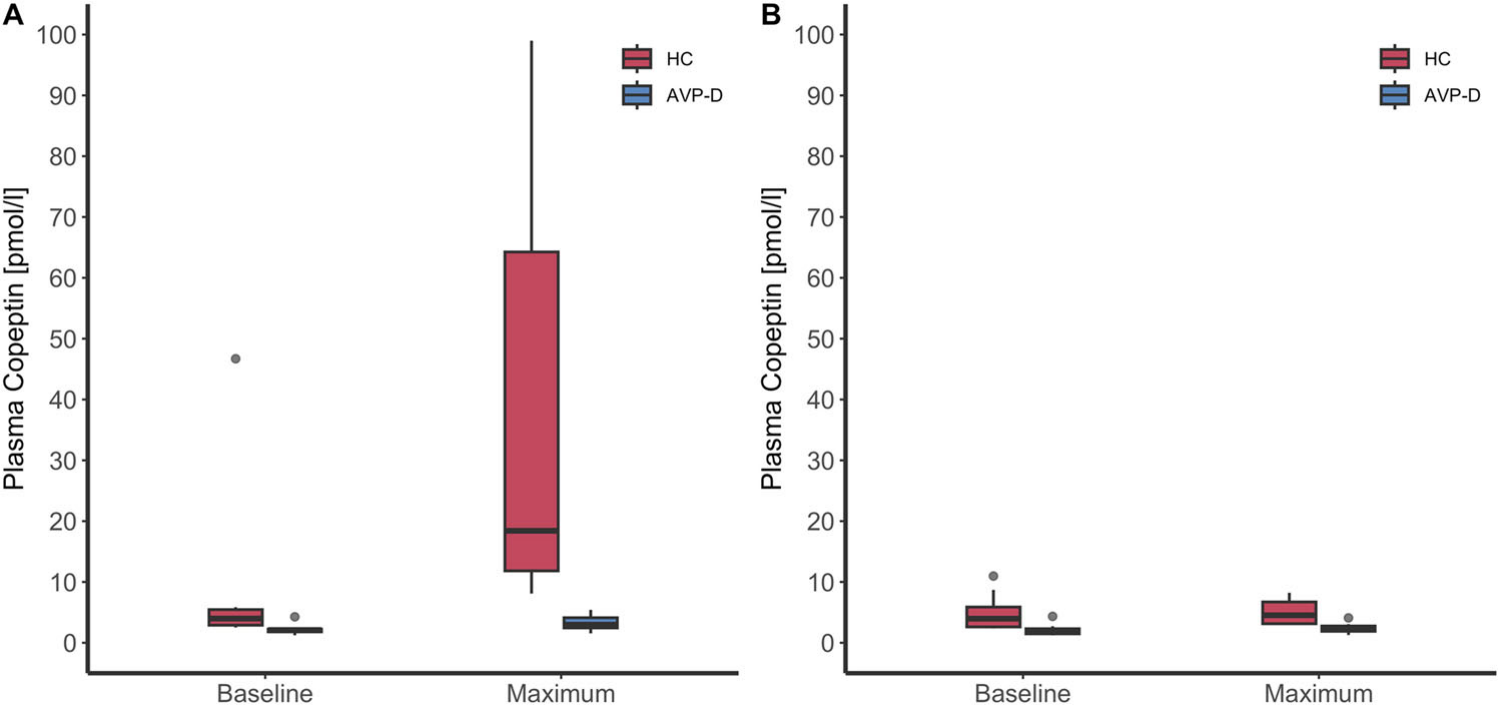
Copeptin in response to glucagon and placebo stimulation. Copeptin levels in pmol/l were visualised with boxplots upon the glucagon stimulation test (**A**) and placebo test (**B**) for ten patients with AVP deficiency (AVP-D) and ten healthy controls (HC)

In healthy participants, the median [IQR] copeptin at baseline was 4.0 pmol/l [2.9–5.5] and increased after injection of glucagon to 18.4 pmol/l [11.8–64.3], resulting in a median increase of 14.0 pmol/l [7.9–46.8]. Under placebo, no notable increase in copeptin was observed: copeptin at baseline was 4.0 pmol/l [2.6–5.9] and remained stable at a copeptin level of 4.5 pmol/l [3.1–6.7], resulting in a median increase of 0.5 pmol/l [–0.5–0.7] (Table 2, Fig. 1). In patients with AVP deficiency, the median copeptin at baseline was 2.1 pmol/l [1.8–2.3] and increased only slightly after glucagon injection to 3.0 pmol/L [2.4–4.1], resulting in a median increase of 0.5 pmol/l [0.2–1.7]. Similarly, under placebo, no notable increase in copeptin was observed: copeptin at baseline was 2.0 pmol/l [1.5–2.3] and remained stable at a copeptin level of 2.2 pmol/l [1.9–2.8], resulting in a median increase of 0.2 pmol/l [0.0–0.4] (Table 2, Fig. 1).

**Table 2:** Laboratory parameters for placebo and glucagon stimulation

	Healthy controls			Patients with AVP-deficiency		
	Placebo	Glucagon	*p* value	Placebo	Glucagon	*p* value
Copeptin at baseline (pmol/l)	4.0 [2.6–5.9]	4.0 [2.9–5.5]	0.85	2.0 [1.5–2.3]	2.1 [1.8–2.3]	0.94
Copeptin maximum (pmol/l)	4.5 [3.1–6.7]	18.4 [11.8–64.3]	<0.01	2.2 [1.9–2.8]	3.0 [2.4–4.1]	0.05
Maximum copeptin change (pmol/l)	0.5 [−0.5 to 0.7]	14.0 [7.9–46.8]	<0.01	0.2 [0.0–0.4]	0.5 [0.2–1.7]	0.14
Oxytocin at baseline (pg/ml)	83.5 [69.3–96.0]	82.7 [62.3–94.3]	0.91	75.7 [70.0–104.1]	73.9 [65.3–81.6]	0.53
Oxytocin maximum (pg/ml)	113.6 [89.4–127.8]	93.3 [87.2–121.1]	0.53	101.8 [88.6–110.0]	114.9 [70.9–140.9]	0.53
Maximum oxytocin change (pg/ml)	25.8 [–1.7–47.3]	24.9 [5.1–27.8]	0.97	13.8 [–5.5 to 31.9]	36.8 [–2.2–51.2]	0.25
Maximum GH change (mIU/l)	–6.6 [–14.3–2.1]	39.3 [7.0–57.4]	<0.01	–2.5 [–7.8–0.8]	0.5 [–10.4–2.6]	0.82
Maximum prolactin change (mIU/l)	–62.5 [–171.8–27.8]	56.0 [13.0–281.0]	0.05	–31.0 [−54.8–(–)3.5]	13.0 [–12.3–73.8]	0.05
Maximum cortisol change (nmol/l)	–296 [–327–(–)215]	165 [−42–04]	<0.01	–106 [−265– (–)13]	190 [–49– 366]	0.01

Data are presented in median [IQR]

*GH* growth hormone, *AVP* arginine vasopressin

**Fig. 2:**
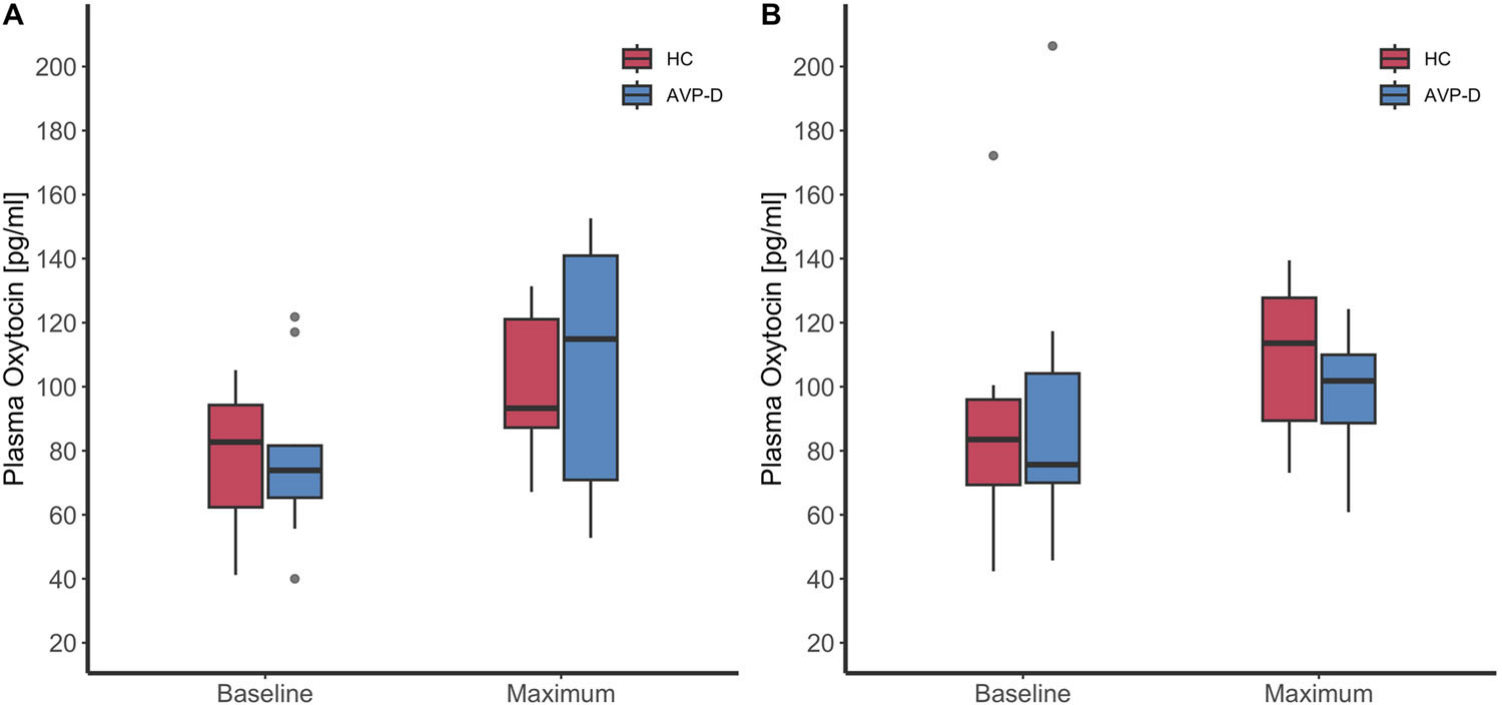
Oxytocin in response to glucagon and placebo stimulation. Oxytocin levels in pg/ml were visualised with boxplots upon the glucagon stimulation test (**A**) and placebo test (**B**) for ten patients with AVP deficiency (AVP-D) and ten healthy controls (HC)

In healthy participants, the median OT at baseline was 82.7 pg/ml [62.3–94.3] and slightly increased to a maximum after injection of glucagon of 93.3 pg/ml [87.2–121.1], resulting in a change increase of 24.9 pg/ml [5.1–27.8]. Under placebo, a comparable increase in OT was observed: OT at baseline was 83.5 [69.3–96.0] and increased to a maximum of 113.6 [89.4–127.8], resulting in a change increase of 25.8 pg/ml [–1.7 –47.3] (Table 2, Fig. 2). Similarly, in patients with AVP deficiency, the median OT at baseline was 73.9 pg/ml [65.3–81.6] and slightly increased after glucagon injection to 114.9 pg/ml [70.9–140.9], resulting in a median increase of 36.8 pg/ml [–2.2–51.2]. Similarly, under placebo, only a slight change in OT was observed: OT at baseline was 75.7 [70.0–104.1] and increased to a maximum of 101.8 [88.6–110.0], resulting in a change increase of 13.8 [–5.5–31.9] (Table 2, Fig. 2).

The mixed model comparing both groups shows that first, there is no effect between glucagon compared to placebo on OT (difference: –0.5 pg/ml; 95%–CI [–25, 24]; *p* = 0.97), and second, that there is no significant treatment-by-group interaction effect, meaning that the effect of glucagon administration was not different in patients with AVP deficiency compared to healthy participants (interaction: 28 pg/ml; 95%–CI [–7, 62]; *p* = 0.13).

### Correlation between decrease in glucose and increase in copeptin and oxytocin levels

**Fig. 3:**
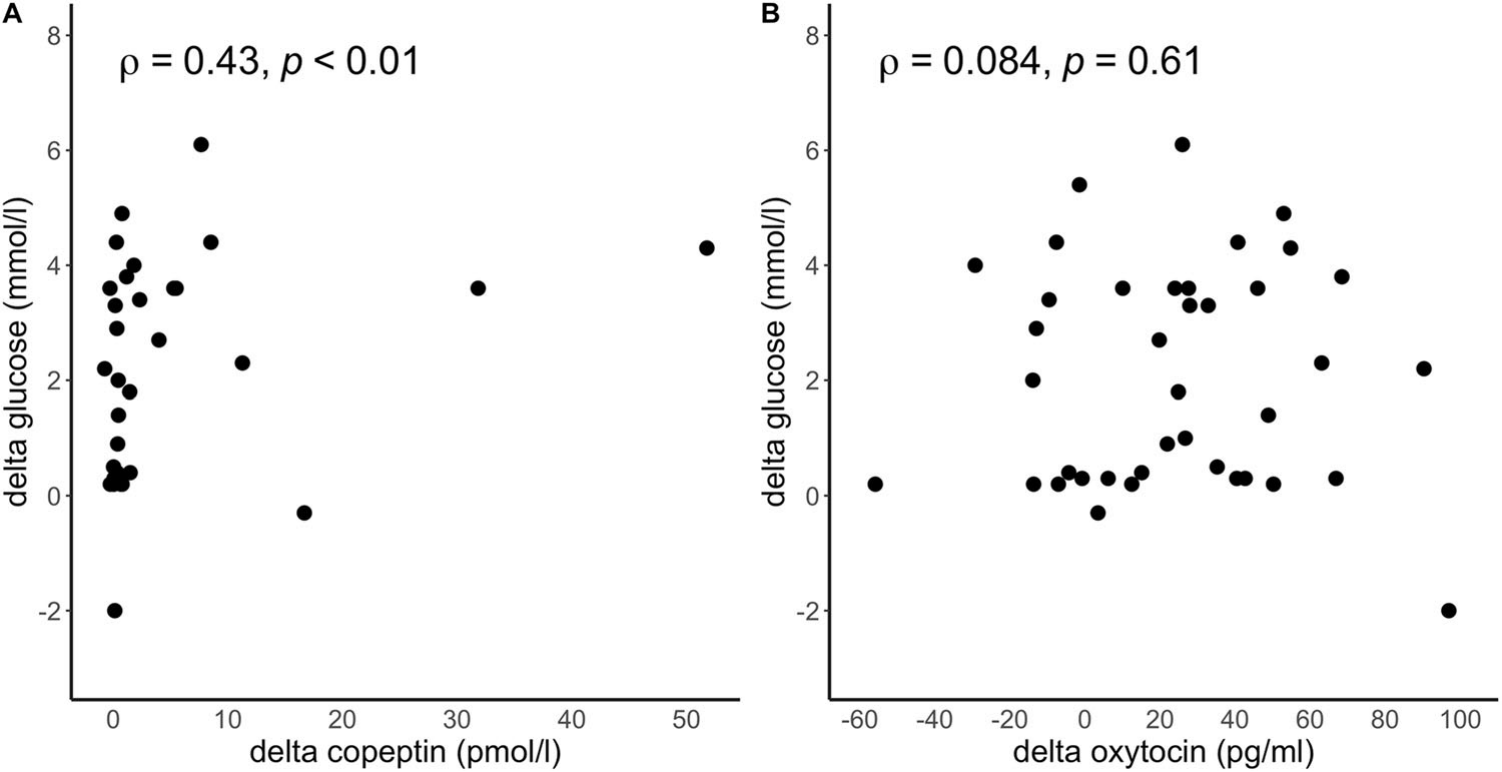
Correlation between glucose and copeptin levels or oxytocin. The delta in the decrease of glucose levels, i.e., maximum glucose—minimum glucose, versus the delta in the increase of copeptin, i.e., maximum copeptin—baseline copeptin (**A**) and versus the delta in the increase of oxytocin, i.e., maximum oxytocin – baseline oxytocin (**B**). Spearman’s rank correlation coefficient ρ is given for each test and the pooled data set

In the pooled dataset, a decrease in glucose levels was significantly correlated with copeptin increase (ρ = 0.43, *p* < 0.01), while the decrease in glucose was not correlated with the change in OT levels (ρ = 0.08, *p* = 0.61) (Fig. 3).

## Discussion

The main finding of our study is that in contrast to a significant increase of copeptin levels upon glucagon, there is no effect of glucagon on plasma OT levels.

Recent data indicated that the social and emotional deficits experienced by patients with AVP deficiency might be explained, at least partly, by an additional OT deficiency [6,8,17–23]. The fact that the observed changes in psychopathology are present in patients with or without additional anterior pituitary dysfunction strengthens this hypothesis [6, 8]. However, measuring and replacing OT is not currently done in clinical routine [19]. Various diagnostic challenges make it difficult to measure basal OT levels accurately and detect a potential deficiency, such as its short half-life in plasma, which lasts only a few minutes [24] and conflicting data regarding whether a single peripheral OT measurement reflects central OT activity [2, 14, 25, 26]. Therefore, an alternative approach, such as provocative testing, might be more suitable to reveal an insufficient OT release [19, 27]. We recently investigated other established pituitary provocation tests, such as the hypertonic saline, the macimorelin or the arginine infusion test, hypothesizing an OT-stimulating effect. Our results, however, indicated that neither of these tests does sufficiently stimulate OT levels in healthy volunteers [28]. MDMA has recently been shown to be a strong stimulus for OT; however, its implication in clinical practice might be challenging. Therefore, an alternative stimulation test is desirable.

Hypoglycaemia strongly stimulates the anterior pituitary, leading to increased levels of growth hormone, cortisol, prolactin and also the posterior pituitary with AVP release [16, 29]. Some studies have indicated that hypoglycaemia induced by the insulin hypoglycaemia test (IHT) also leads to OT release [10, 11]. Chiodera et al. have shown a doubling in plasma OT levels in healthy volunteers following 30 min of profound hypoglycaemia. Mechanistically, it is postulated that OT is involved in the central regulation of pancreatic secretion and its release might be stimulated by brain-glucose-sensing neurons located in the hypothalamus and can be activated by the sympathetic autonomic branch [15]. We recently provided evidence for a strong effect of glucagon on plasma copeptin levels as a surrogate marker of AVP [12]. One possible explanation for this stimulating effect might be the dynamics of glucose levels after glucagon injection. Initially, plasma glucose levels increase from baseline and reach a peak, followed by a rapid decrease, bringing glucose levels back to low or normal levels [9]. We demonstrated a clear correlation between the drop in glucose levels and the increase in copeptin levels upon glucagon stimulation supporting this hypothesis [9]. Therefore, it is possible that the rapid decrease in elevated glucose levels might mimic – in analogy to IHT – an acute relative hypoglycaemic state without inducing absolute hypoglycaemia [9]. However, although it seems that this postulated effect of relative hypoglycaemia is potent enough to stimulate copeptin, it appears that OT is only stimulated by absolute hypoglycaemia, for which glucagon is not a strong enough stimulus. Alternatively, the hypothesised effect of glucagon might have been attenuated by concurrent administration of ondansetron, a serotonin (5-HT) receptor antagonist. In support of this, animal studies indicate that 5-HT and receptor agonists stimulate AVP and OT release [30, 31]. Conversely, in 1998, Volpi et al. demonstrated in a study of 12 participants that ondansetron partially reduced AVP response to hypoglycaemia but interestingly not OT response [32]. However, due to the limited sample size the possibility of ondansetron exerting a similar suppressive release on OT cannot be completely excluded. Moreover, as we only measured OT up to 120 min after glucagon injection, contrary to 180 min in the study of Volpi et al., we could have missed the stimulatory effect. Furthermore, despite the hypothesis of a glucagon-induced relative hypoglycaemia-mediated OT effect, the exact mechanisms remain unclear. Some previous research also discussed stimulating active peptides fragmented directly from glucagon [33, 34]. In support of this, various application routs of glucagon have been shown to stimulate the pituitary gland differently [35]. Currently, further agents are under investigation for OT stimulation, i.e., GLP-1 analogues, melatonin, and corticotropin-releasing-hormone (clinicaltrials.gov NCT04897802, NCT04902235).

Considering the so far negative results on OT levels for most stimulation tests, and taking into account the strong stimulus, MDMA remains currently the most promising test to diagnose possible OT deficiency. As MDMA is currently in phase 3 trials for the development as a therapeutic agent for post-traumatic stress disorders, the use of MDMA as a diagnostic agent will be directly accessible to clinicians in the near future. Therefore, further developing the MDMA test seems to be the most promising method for clinical routine. Moreover, for clinical practice, addressing preanalytical and analytical challenges associated with OT measurements are crucial before implementing diagnostic tests. The reliability of currently available assays, including EIA and RIA, remains a subject of ongoing debate. Consensus among experts emphasizes standardized analytical procedures to preserve accuracy and reproducibility, entailing cooled plasma samples, batch measurement, and sample extraction—methods followed in our study [36]. Nevertheless, as promising methods (liquid chromatography/mass spectrometry, LC/MS) are in development, available assays should be used with their respective limitations. Consequently, as suggested, OT results should be interpreted not in absolute terms but rather in relation to relative changes [37].

Some limitations should be considered for our study. First, this is a secondary analysis of a prospective diagnostic study. Despite careful blood sampling, immediate centrifugation, and quick storage at –80 °C, a certain decay of OT cannot be excluded, presenting a certain bias as the OT assay requires standardized procedures in blood sampling and storage. Considering the poor response of OT to glucagon stimulation, the limited sensitivity of the test and its challenging handling characteristics might have influenced the findings of the study. Second, the sample size was limited and did not allow for sub-group analysis. Third, although all samples were measured in one batch, there is ongoing discussion on the currently available OT enzyme immunoassay (EIA) and radioimmunoassay (RIA). In conclusion, our study demonstrates that glucagon does not stimulate OT levels.
